# Weight cycling and risk of clinical adverse events in patients with heart failure with preserved ejection fraction: a *post-hoc* analysis of TOPCAT

**DOI:** 10.3389/fendo.2023.1159826

**Published:** 2023-05-10

**Authors:** Yi Tan, Hang Guo, Ning Zhang, Keyang Zheng, Guifang Liu

**Affiliations:** ^1^ Department of Education, Beijing Chaoyang Hospital, Capital Medical University, Beijing, China; ^2^ Department of Education, Beijing Anzhen Hospital, Capital Medical University, Beijing, China; ^3^ Department of Education, Beijing Stomatological Hospital, Capital Medical University, Beijing, China; ^4^ Department of Radiology, Qingdao Central Hospital, Qingdao, Shandong, China

**Keywords:** body mass index, waist circumference, heart failure with preserved ejection fraction, weight cycling, diabetes mellitus

## Abstract

**Background:**

Previous studies hardly evaluated the association of variability of body mass index (BMI) or waist circumference with clinical adverse events and investigated whether weight cycling had an effect on the prognosis of patients with heart failure with preserved ejection fraction (HFpEF).

**Methods:**

This study was a *post-hoc* analysis of TOPCAT. Three outcomes were evaluated: the primary endpoint, cardiovascular disease (CVD) death, and heart failure hospitalization. Among them, CVD death and hospitalization were outcomes of heart failure. Kaplan–Meier curves were used to describe the cumulative risk of outcome and were tested using the log-rank test. Cox proportional hazards regression models were used to calculate hazard ratios (HRs) and 95%CIs for outcomes. We also performed a subgroup analysis, and several subgroups were compared.

**Results:**

A total of 3,146 patients were included. In the Kaplan–Meier curves, the coefficients of variation of both BMI and waist circumference were grouped according to quartiles, with the Q4 group having the highest cumulative risk (log-rank *P* < 0.001). In the coefficient of BMI variation and the outcomes, the HRs for group Q4 of coefficient of variation of BMI were 2.35 (95%CI: 1.82, 3.03) for the primary endpoint, 2.40 (95%CI: 1.69, 3.40) for death, and 2.33 (95%CI: 1.68, 3.22) for HF hospitalization in model 3 (fully adjusted model) compared with group Q1. In the coefficient of waist circumference variation and the outcomes, group Q4 had increased hazard of the primary endpoint [HR: 2.39 (95%CI: 1.84, 3.12)], CVD death [HR: 3.29 (95%CI: 2.28, 4.77)], and HF hospitalization [HR: 1.98 (95%CI 1.43, 2.75)] in model 3 (fully adjusted model) compared with group Q1. In the subgroup analysis, there was a significant interaction in the diabetes mellitus subgroup (*P* for interaction = 0.0234).

**Conclusion:**

Weight cycling had a negative effect on the prognosis of patients with HFpEF. The presence of comorbid diabetes weakened the relationship between waist circumference variability and clinical adverse events.

## 1 Introduction

Obesity is common in heart failure with preserved ejection fraction (HFpEF) patients and is considered as an independent risk factor for the development of HFpEF (1). Thus, weight management is important for HFpEF patients with obesity. A single central trial showed that a modest weight loss could improve the exercise capacity and quality of life for obesity and HFpEF patients (2). There is also evidence that weight loss in obesity can improve left ventricular concentric remodeling in patients with heart failure (3).

However, overweight and obese individuals are likely to result in an equal or greater weight gain after the resultant weight loss with a poor weight loss method, which is called weight cycling (4). Weight cycling has been shown to correlate with adverse events in diabetic populations (5) and coronary artery disease (CAD) (6) populations. Moreover, recent studies have identified weight cycling as a risk factor for cardiometabolic diseases independent of body weight (7).

The variability of body mass index (BMI) and waist circumference are two indicators that show the fluctuation in body weight and can reflect the process of weight cycling. Since BMI is a parameter reflecting overall body weight and waist circumference, as another parameter reflecting abdominal obesity, it may be more associated with adverse cardiovascular disease (CVD) outcomes (8, 9). Many studies have focused on the relationship between changes in BMI and a poor prognosis of CVD. However, in patients with HFpEF, no clinical studies have focused on the prognostic impact of variability in BMI or waist circumference. Thus, we conducted a *post-hoc* analysis of TOPCAT to evaluate the relationship of the variability of BMI or waist circumference on the prognosis of HFpEF. This study aimed to investigate whether such weight cycling has an effect on the prognosis of patients with HFpEF.

## 2 Method

### 2.1 Study design and population

This study is a *post-hoc* analysis of TOPCAT. The data was obtained from the BioLINCC website (https://biolincc.nhlbi.nih.gov/), and we were licensed to use this data. TOPCAT is a randomized, double-blind trial of patients with symptomatic heart failure and ejection fraction of 45% or greater. Adverse events such as death and hospitalization due to heart failure were compared between treatment with spironolactone (15 to 45 mg per day) and placebo (10). The study enrolled 3,445 patients who met the criteria and were followed for 3.3 years (mean follow-up time). The original study found no significant difference in outcomes between the two groups of patients receiving different treatments.

**Figure 1:**
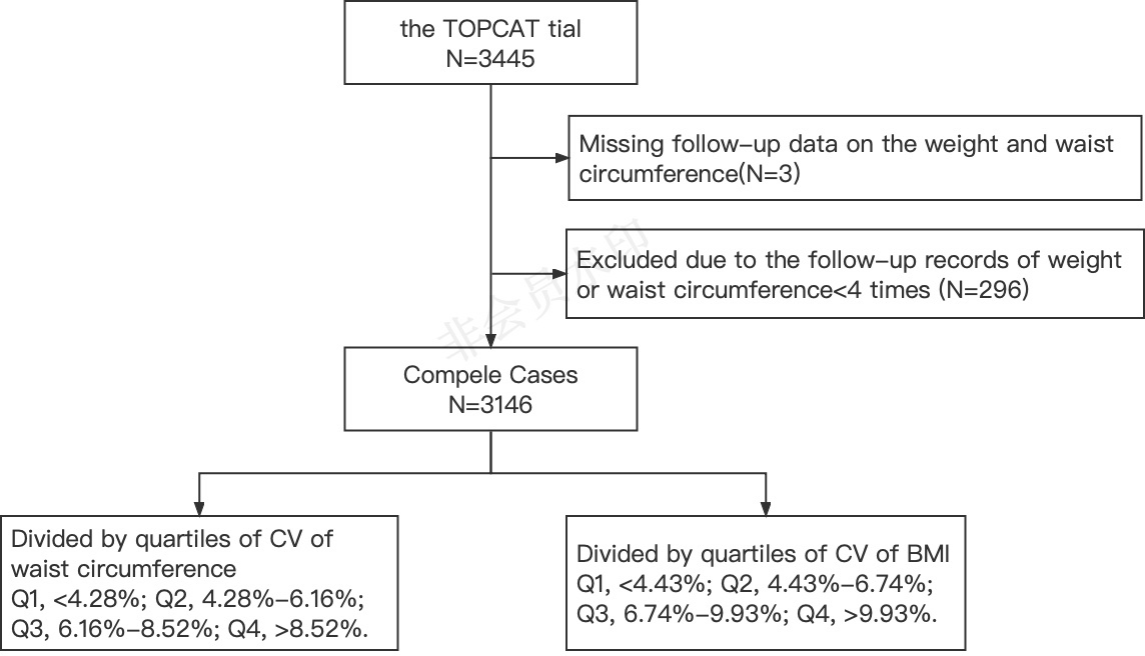
Flow chart for enrolling patients in this study.

In the analysis of this study, patients were included according to the following steps: In the first step, three patients who were not followed up for weight and waist circumference were excluded; secondly, among the patients who completed the follow-up, 296 patients with fewer than four follow-up records of weight or waist circumference were excluded. Finally, 3,146 patients were included in this study (**Figure 1**).

### 2.2 Assessment of variability of BMI and waist circumference and outcome

Height data were collected at baseline to calculate the BMI. The patients were followed up for weight and waist circumference at 4 weeks, 8 weeks, 4 months, and every 4 months thereafter after enrollment. Considering that the traditional description variable standard deviation (SD) excluded the influence of mean value, the variability of BMI and waist circumference is thus described by coefficients of variation (CV).

Three outcomes were evaluated in this study (1): primary endpoint of the original study, (2) CVD death, and (3) heart failure (HF) hospitalization. The definition of the primary endpoint is cardiovascular mortality, aborted cardiac arrest, or hospitalization for the management of heart failure as a composite. More definitions of outcomes can be found in the original study (10).

### 2.3 Statistical analysis

The patients’ characteristics were presented in quartiles of the CV of waist circumference. Continuous variables were expressed as mean (SD) or median (IQR), and ANOVA or Kruskal–Wallis test was used to compare differences between groups; categorical variables were expressed as proportions, and *χ* ^2^ test was used for comparison between groups.

Kaplan–Meier (KM) curves were used to describe the cumulative risk of outcome between different BMI or waist circumference variability groups and were tested using log-rank test.

Cox proportional hazards regression models were used to calculate hazard ratios (HRs) and 95%CIs for outcomes (primary endpoint, death, and HF hospitalization). The CV of waist circumference or BMI was assessed as categorical variables. In this study, three models were used for waist circumference or BMI: model 1 adjusted for none; model 2 adjusted for age, sex, and race; and model 3 included model 2 plus adjustment for current smoking, intervention, NYHA class, diabetes mellitus, glucose, eGFR, and baseline medication.

The subgroup analysis was performed using model 3 (covariates that were tested for interactions were excluded), and we treated the CV of BMI or waist circumference as a continuous variable (per group). The following subgroups were compared: male vs. female; age <69 vs. age ≥69; White vs. Black vs. others; visit-to-visit mean BMI <30 vs. visit-to-visit mean BMI ≥30; visit-to-visit mean waist circumference <101.3 vs. visit-to-visit mean waist circumference ≥101.3; and with diabetes mellitus vs. without diabetes mellitus.

All statistical analyses were performed using R version 4.2.2 (Vienna, Austria, https://www.r-project.org/). Bilateral *P <*0.05 was considered statistically significant.

## 3 Results

### 3.1 Characteristics of the enrolled patients

A total of 3,146 patients were included, with a mean age of 68.4 ± 9.5 years old and with 1,536 (48.8%) being male patients. The patients were divided into four groups according to the quartiles of CV of waist circumference, and the characteristics of the patients are shown in **Table 1**. Compared with group Q1, group Q4 was older, more likely to be female patients, less likely to be White, and less likely to smoke. Regarding medical history, group Q1 had the highest prevalence of DM and angina, and group Q2 had the highest history of past myocardial infarction. Regarding laboratory tests, Q4 group had the lowest blood glucose levels and EGFR. At baseline, the utilization rate of ACEI/ARB and beta receptor antagonist in group Q4 was the lowest. The visit-to-visit mean BMI and waist circumference of the Q4 group were both the lowest. Regarding outcomes, Q4 group had the highest rate of the primary endpoint, CVD death, and HF hospitalization.

**Table 1:** Characteristics of 3,146 patients by quartiles of coefficient of variation of waist circumference.

Characteristics	Q1, <4.28% *N* = 787	Q2,4.28%–6.16% *N* = 786	Q3, 6.16%–8.52% *N* = 786	Q4, >8.52% *N* = 787	*P*-value
Age, years	64.88 ± 8.50	67.23 ± 9.15	69.00 ± 9.27	72.41 ± 9.53	<0.001
Sex					<0.001
Male, *n* (%)	506 (64.29%)	428 (54.45%)	368 (46.82%)	234 (29.73%)	
Female, *n* (%)	281 (35.71%)	358 (45.55%)	418 (53.18%)	553 (70.27%)	
Race					<0.001
White, *n* (%)	721 (91.61%)	723 (91.98%)	719 (91.48%)	685 (87.04%)	
Black, *n* (%)	58 (7.37%)	57 (7.25%)	47 (5.98%)	66 (8.39%)	
Others, *n* (%)	8 (1.02%)	6 (0.76%)	20 (2.54%)	36 (4.57%)	
Current smoking, *n* (%)	96 (12.20%)	85 (10.81%)	87 (11.07%)	63 (8.01%)	0.047
Visit-to-visit mean BMI, kg/m^2^	34.78 ± 7.25	32.82 ± 6.14	31.92 ± 6.35	30.40 ± 6.10	<0.001
Visit-to-visit mean waist circumference, cm	108.35 ± 18.81	104.37 ± 16.08	101.96 ± 14.55	97.03 ± 14.64	<0.001
Intervention, *n* (%)	394 (50.06%)	379 (48.22%)	409 (52.04%)	396 (50.32%)	0.512
Medical history					
Hypertension, *n* (%)	729 (92.63%)	724 (92.11%)	716 (91.09%)	710 (90.22%)	0.319
Diabetes mellitus, *n* (%)	296 (37.61%)	234 (29.77%)	223 (28.37%)	242 (30.75%)	<0.001
Angina, *n* (%)	433 (55.02%)	407 (51.78%)	362 (46.06%)	317 (40.28%)	<0.001
History of myocardial infarction, *n* (%)	198 (25.16%)	248 (31.55%)	194 (24.68%)	193 (24.52%)	0.003
History of stroke, *n* (%)	53 (6.73%)	66 (8.40%)	60 (7.63%)	58 (7.37%)	0.658
NYHA class					0.669
I–II class, *n* (%)	534 (67.85%)	537 (68.32%)	554 (70.48%)	537 (68.23%)	
III–IV class, *n* (%)	253 (32.15%)	249 (31.68%)	232 (29.52%)	250 (31.77%)	
Laboratory tests					
Glucose, mg/dl, median (Q1–Q3)					
Class 1	98.18 (89.09–109.09)	98.00 (89.09–107.27)	97.00 (88.00–109.00)	98.18 (89.09–107.27)	0.608
Class 2	120.00 (100.00–140.45)	114.55 (105.09–125.91)	114.55 (101.82–144.00)	121.00 (100.00–129.02)	0.624
Class 3	144.00 (109.09–181.00)	128.00 (103.32–170.45)	134.55 (109.00–185.45)	130.50 (102.41–177.00)	0.463
eGFR, ml/(min·1.73m^2^)	69.71 ± 19.54	68.83 ± 19.47	67.65 ± 19.82	65.48 ± 19.70	<0.001
Baseline medication					
ACEI/ARB, *n* (%)	712 (90.47%)	654 (83.21%)	656 (83.46%)	643 (81.81%)	<0.001
Beta receptor antagonist, *n* (%)	619 (78.65%)	638 (81.17%)	603 (76.72%)	594 (75.57%)	0.041
Calcium channel blockers, *n* (%)	296 (37.61%)	283 (36.01%)	312 (39.69%)	290 (36.90%)	0.478
Diuretic agent, *n* (%)	663 (84.24%)	639 (81.30%)	639 (81.30%)	627 (79.77%)	0.138
Outcomes					
Primary endpoint, *n* (%)	104 (13.21%)	128 (16.28%)	149 (18.96%)	187 (23.76%)	<0.001
CVD death, *n* (%)	70 (8.89%)	91 (11.58%)	107 (13.61%)	171 (21.73%)	<0.001
Heart failure hospitalization, *n* (%)	289 (36.72%)	347 (44.15%)	356 (45.29%)	401 (50.95%)	<0.001

Class 1 means glucose for patients without diabetes mellitus (DM), class 2 means glucose for DM patients without anti-diabetic medications, and class 3 means glucose for DM patients with anti-diabetic medications.

### 3.2 KM curves of BMI or waist circumference variability and outcomes

**Figure 2:**
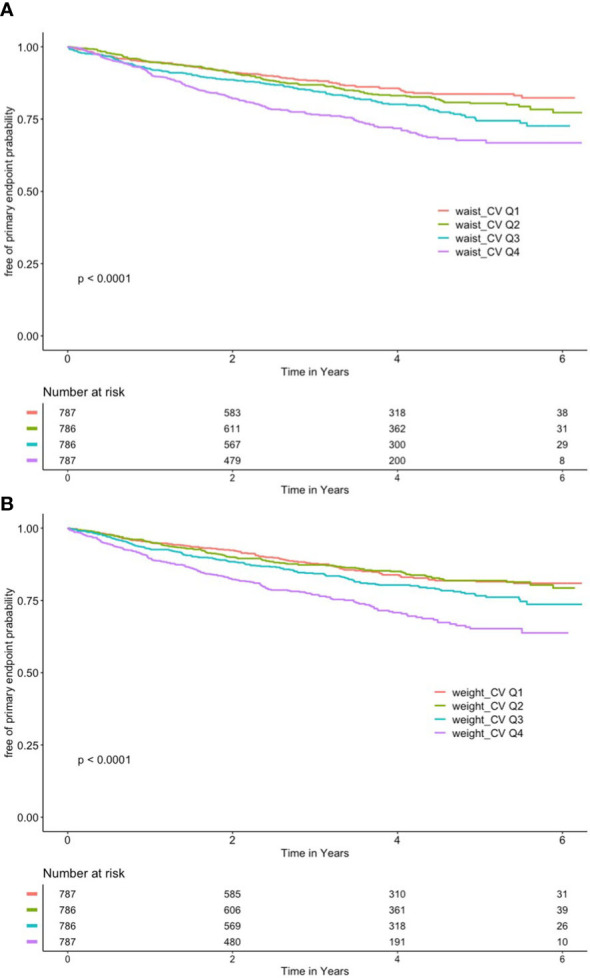
**(A)** Kaplan–Meier curve of different coefficient variations of waist and primary outcome. **(B)** Kaplan–Meier curve of different coefficient variations of body mass index and primary outcome.

As shown in **Figure 2A**, after grouping according to quartiles of CV of waist circumference, the cumulative risk of the primary endpoint was significantly different among the four groups, with the highest cumulative risk in the Q4 group (log-rank *P* < 0.001).

As shown in **Figure 2B**, the CV of BMI was also grouped according to quartiles, with the Q4 group having the highest cumulative risk (log-rank *P* < 0.001).

### 3.3 Coefficient of variation of waist circumference and outcomes

Hazard ratios for outcomes by CV of waist circumference are shown in **Table 2**. Group Q4 had increased hazard of the primary endpoint [HR: 2.39 (95%CI: 1.84, 3.12)], CVD death [HR: 3.29 (95%CI: 2.28, 4.77)], and HF hospitalization [HR 1.98 (95%CI: 1.43, 2.75)] in model 3 (fully adjusted model) compared with group Q1. *P* for trend was also calculated in all outcomes, and the trend was all significant in model 3.

**Table 2:** Hazard ratios for outcomes by coefficient of variation of waist circumference.

Outcomes	Q1, <4.28%	Q2, 4.28%–6.16%	Q3, 6.16%–8.52%	Q4, >8.52%	*P* for trend
Primary endpoint Hazard ratios (95%CI), *P*-value					
Model 1	Reference	1.17 (0.90, 1.51) 0.2369	1.47 (1.15, 1.89) 0.0024	1.17 (0.90, 1.51) 0.1613	<0.0001
Model 2	Reference	1.17 (0.90, 1.52) 0.2358	1.50 (1.16, 1.94) 0.0018	2.10 (1.62, 2.73) 0.0002	<0.0001
Model 3	Reference	1.21 (0.93, 1.57) 0.1613	1.64 (1.26, 2.12) 0.0002	2.39 (1.84, 3.12) <0.0001	<0.0001
CVD death Hazard ratios (95%CI), *P*-value					
Model 1	Reference	0.99 (0.66, 1.46) 0.9430	1.53 (1.06, 2.21) 0.0217	3.00 (2.14, 4.21) <0.0001	<0.0001
Model 2	Reference	1.00 (0.67, 1.48) 0.9911	1.54 (1.06, 2.24) 0.0224	3.17 (2.20, 4.57) <0.0001	<0.0001
Model 3	Reference	1.02 (0.69, 1.53) 0.9040	1.60 (1.10, 2.33) 0.0136	3.29 (2.28, 4.77) <0.0001	<0.0001
Heart failure hospitalization Hazard ratios (95%CI), *P*-value					
Model 1	Reference	1.31 (0.96, 1.79) 0.0833	1.45 (1.07, 1.97) 0.0177	1.84 (1.36, 2.48) <0.0001	<0.0001
Model 2	Reference	1.28 (0.94, 1.75) 0.1182	1.42 (1.04, 1.95) 0.0267	1.64 (1.19, 2.26) 0.0027	<0.0001
Model 3	Reference	1.31 (0.95, 1.79) 0.0958	1.60 (1.17, 2.20) 0.0035	1.98 (1.43, 2.75) <0.0001	<0.0001

Model 1 adjusted for none. Model 2 adjusted for age, sex, and race. Model 3 adjusted for age, sex, race, current smoking, intervention, NYHA class, diabetes mellitus, glucose, eGFR, and baseline medication.

### 3.4 Coefficient of variation of BMI and outcomes

Hazard ratios for outcomes by BMI of waist circumference are shown in **Table 3**. The HRs for group Q4 of coefficient of variation of BMI were 2.35 (95%CI: 1.82, 3.03) for the primary endpoint, 2.40 (95%CI: 1.69, 3.40) for death, and 2.33 (95%CI: 1.68, 3.22) for HF hospitalization in model 3 (fully adjusted model) compared with group Q1. *P* for trend was <0.001 in all outcomes in the fully adjusted model (model 3).

**Table 3:** Hazard ratios for outcomes by coefficient of variation of body mass index.

Outcomes	Q1, <4.43%	Q2, 4.43%–6.73%	Q3, 6.73%–9.93%	Q4, >9.93%	*P* for trend
Primary endpoint Hazard ratios (95%CI), *P*-value					
Model 1	Reference	1.02 (0.79, 1.33) 0.8569	1.33 (1.04, 1.71) 0.0222	2.07 (1.64, 2.61) <0.0001	<0.0001
Model 2	Reference	1.06 (0.82, 1.37) 0.6682	1.32 (1.03, 1.70) 0.0286	2.05 (1.59, 2.63) <0.0001	<0.0001
Model 3	Reference	1.12 (0.86, 1.46) 0.3813	1.41 (1.10, 1.83) 0.0078	2.35 (1.82, 3.03) <0.0001	<0.0001
CVD death Hazard ratios (95%CI), *P*-value					
Model 1	Reference	0.82 (0.56, 1.19) 0.2875	1.09 (0.77, 1.56) 0.6181	2.37 (1.72, 3.26) <0.0001	<0.0001
Model 2	Reference	0.82 (0.56, 1.20) 0.3092	1.05 (0.73, 1.51) 0.7841	2.28 (1.62, 3.21) <0.0001	<0.0001
Model 3	Reference	0.84 (0.57, 1.23) 0.3682	1.09 (0.76, 1.57) 0.6481	2.40 (1.69, 3.40) <0.0001	<0.0001
Heart failure hospitalization Hazard ratios (95%CI), *P*-value					
Model 1	Reference	1.22 (0.89, 1.68) 0.2227	1.51 (1.11, 2.05) 0.0086	2.07 (1.54, 2.79) <0.0001	<0.0001
Model 2	Reference	1.27 (0.92, 1.74) 0.1474	1.46 (1.07, 2.00) 0.0173	1.94 (1.42, 2.66) <0.0001	<0.0001
Model 3	Reference	1.33 (0.96, 1.83) 0.0864	1.56 (1.14, 2.15) 0.0058	2.33 (1.68, 3.22) <0.0001	<0.0001

Model 1 adjusted for none. Model 2 adjusted for age, sex, and race. Model 3 adjusted for age, sex, race, current smoking, intervention, NYHA class, diabetes mellitus, glucose, eGFR, and baseline medication.

### 3.5 Subgroup analysis of variability of BMI or waist circumference and outcomes

**Figure 3:**
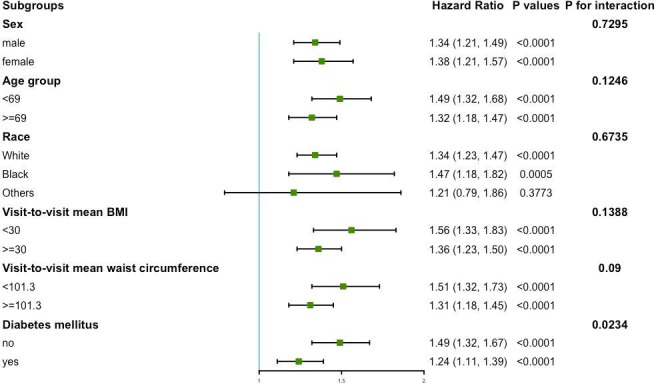
Subgroup analysis for the risk of primary endpoint by coefficient of variation of waist circumference (per group). Model 3 was used, but the covariates that were tested for interactions were excluded.

The results of the subgroup analysis of variability of waist circumference and primary endpoint are shown in **Figure 3**. No significant interactions were found in sex, age, race, mean BMI, and mean waist circumference subgroups (all *P* for interaction >0.05). However, this study found a significant interaction in the diabetes mellitus subgroup (*P* for interaction = 0.0234). In the diabetic population, the HR per group increase in the CV of waist circumference was 1.24 (95%CI: 1.11, 1.39) for the primary endpoint. In the population without diabetes mellitus, the HR per group increase in the CV of waist circumference was 1.49 (95%CI: 1.32, 1.67) for the primary endpoint.

**Figure 4:**
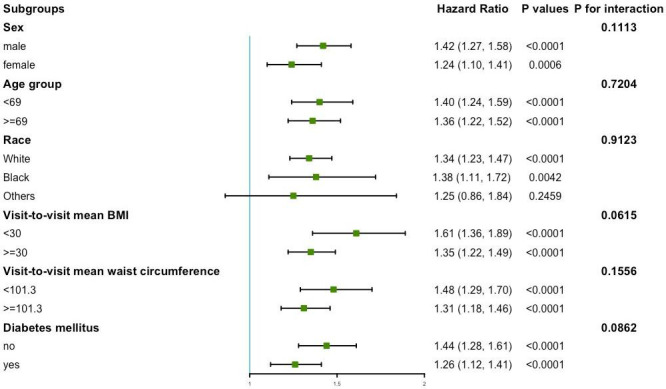
Subgroup analysis for the risk of primary endpoint by coefficient of variation of body mass index (per group). Model 3 was used, but the covariates that were tested for interactions were excluded.

**Figure 4** presents the variability of BMI and the results of the subgroup analysis of the primary endpoint. No significant interactions were found in sex, age, race, mean BMI, mean waist circumference, and diabetes mellitus subgroups (all *P* for interaction >0.05).

## 4 Discussion

This study evaluated the relationship between weight cycling and clinical outcomes in HFpEF patients. Both BMI variability and waist circumference variability were found to be significantly associated with clinical adverse events, and the risk of clinical adverse events increased with increasing variability. In the subgroup analysis, we found that the presence or absence of comorbid diabetes affected the relationship between waist circumference variability and clinical adverse events.

Previous studies have also discussed the association between weight cycling and clinical adverse events in people with type 2 diabetes. Arnaud D. Kaze conducted a prospective cohort study and found a positive and consistent correlation between weight cycling and CVD outcomes and deaths in people with type 2 diabetes (5). Moreover, the study also researched whether an intensive lifestyle intervention can affect this association. An intensive lifestyle intervention had the purpose of 7% weight loss or greater via more physical activity and less caloric intake compared with the standard of care (diabetes support and education). The outcomes showed that an intensive lifestyle intervention could alter the association of weight cycling with cardiovascular outcomes and deaths, which provided a possible opportunity for people with type 2 diabetes to eliminate the impacts of weight cycling when losing weight. However, a report from the NHLBI-sponsored WISE Study found an opposite outcome about the association between weight cycling and clinical adverse events in people with suspected ischemia (6). In their research, weight cycling was associated with a lower cardiovascular outcome rate in women with suspected ischemia despite the influence of HDL-cholesterol. The findings were not consistent with prior studies in men (11). The possible reason might be the sex differences in metabolism, fat storage, diabetes, and CVD.

The mechanisms underlying the association between variability in BMI or waist circumference and poor prognosis are not yet fully understood. One possible mechanism is that fluctuations in body weight lead to changes in fat metabolism. It has been found that when the body loses weight (especially when dieting to lose weight), the participating lipocytes will grow and proliferate more rapidly if the body gains weight again afterwards, probably because the metabolic shift tends to be more favorable for lipid accumulation (12, 13). It has also been found that repeated weight loss and then recovery preferentially promotes the gain of abdominal fat and is associated with a poor prognosis of cardiovascular disease (13, 14). This is also similar to our finding that the variability of waist circumference, which better represents abdominal fat, was associated with a higher risk of death from CVD than the variability of BMI. Another possible mechanism is the adipocyte-associated inflammatory response. The rapid remodeling of adipose tissue associated with weight fluctuations may lead to the abnormal production of pro-inflammatory factors (15), and weight fluctuations have been found to be associated with elevated circulating C-reactive protein concentrations which had adverse effects on the cardiovascular system. Animal studies have also found an overproduction of lymphocytes and a large increase in cytokines in the adipose tissue of weight-cycling mice, which may be a possible mechanism by which the inflammatory response mediates weight cycling and poor prognosis (14).

Our study also found that the presence or absence of diabetes affected the relationship between waist circumference variability and the primary endpoint, with the relationship between the two attenuated by the presence of diabetes. Based on previous studies, one of the possible mechanisms for the relationship was the altered metabolism of adipocytes. Previous studies have found that fat metabolism is abnormal in DM patients, with reduced glucose uptake and lipolysis in adipocytes from DM patients, and the extracellular matrix from DM patients has been found to impair glucose uptake in adipocytes from non-DM patients (16). It has also been suggested that it may be that adipocytes promote the formation of insulin resistance in patients with DM, thus affecting the metabolism of the organism (17). In addition to the mechanisms mentioned above, more metabolic disorders were also found in patients with DM (18), which may attenuate the relationship between variability in waist circumference and adverse events.

Our study has strength in such a way that we introduced the concept of weight cycling into the evaluation of the prognosis of HFpEF since previous studies paid little attention to the variability of the BMI and waist circumference. Moreover, the TOPCAT study had a long follow-up time of 3.3 years which helped to observe the fluctuation of such two slowly changing indicators.

We acknowledged that there were some limitations to our study. Since it was a *post-hoc* analysis of a trial, it could not conform to the population and the randomization model of statistical inference. The explicit mechanisms of the effect of weight cycling on the poor prognosis of cardiovascular disease are also unclear and remain to be verified.

## 5 Conclusion

In this *post-hoc* analysis of TOPCAT, we found that weight cycling had a negative effect on the prognosis of patients with HFpEF. The presence of comorbid diabetes weakened the relationship between waist circumference variability and clinical adverse events. These findings indicated BMI and waist circumference as independent risk factors for clinical adverse events. When losing weight, it is important for patients with HFpEF to pay attention to weight cycling and take action to smoothen the fluctuations of BMI and waist circumference. We look forward to more effective weight loss methods to prevent weight cycling and maintain a long-term effect of weight loss, which could further reduce the risk of patients with heart failure with preserved ejection fraction.

## Data availability statement

Publicly available datasets were analyzed in this study. This data can be found here: the BioLINCC website (https://biolincc.nhlbi.nih.gov/).

## Ethics statement

The studies involving human participants were reviewed and approved by the Institutional Review Committee of Beijing Chaoyang Hospital. The patients/participants provided their written informed consent to participate in this study.

## Author contributions

YT wrote the manuscript. YT, HG, and KZ applied for the database and performed the statistical analysis. NZ and GL revised the manuscript. All authors contributed to the article and approved the submitted version.
